# Quantitative assessment of intragenic receptor tyrosine kinase deletions in primary glioblastomas: their prevalence and molecular correlates

**DOI:** 10.1007/s00401-013-1217-3

**Published:** 2013-11-29

**Authors:** Edward R. Kastenhuber, Jason T. Huse, Samuel H. Berman, Alicia Pedraza, Jianan Zhang, Yoshiyuki Suehara, Agnes Viale, Magali Cavatore, Adriana Heguy, Nicholas Szerlip, Marc Ladanyi, Cameron W. Brennan

**Affiliations:** 1 Department of Pathology, Memorial Sloan-Kettering Cancer Center, New York, NY USA; 2 Human Oncology and Pathogenesis Program, Memorial Sloan-Kettering Cancer Center, New York, NY USA; 3 Brain Tumor Center, Memorial Sloan-Kettering Cancer Center, New York, NY USA; 4 Genomics Core Facility, Memorial Sloan-Kettering Cancer Center, New York, NY USA; 5 Geoffrey Beene Translational Oncology Core Facility, Memorial Sloan-Kettering Cancer Center, New York, NY USA; 6 Department of Neurosurgery, Wayne State University Medical School, Detroit, MI USA; 7 Department of Neurosurgery, Memorial Sloan-Kettering Cancer Center, New York, NY USA

**Keywords:** EGFRvIII, GBM, Glioblastoma, Nanostring, RNA sequencing, TCGA

## Abstract

Intragenic deletion is the most common form of activating mutation among receptor tyrosine kinases (RTK) in glioblastoma. However, these events are not detected by conventional DNA sequencing methods commonly utilized for tumor genotyping. To comprehensively assess the frequency, distribution, and expression levels of common RTK deletion mutants in glioblastoma, we analyzed RNA from a set of 192 glioblastoma samples from The Cancer Genome Atlas for the expression of EGFRvIII, EGFRvII, EGFRvV (carboxyl-terminal deletion), and PDGFRAΔ8,9. These mutations were detected in 24, 1.6, 4.7, and 1.6 % of cases, respectively. Overall, 29 % (55/189) of glioblastomas expressed at least one RTK intragenic deletion transcript in this panel. For EGFRvIII, samples were analyzed by both quantitative real-time PCR (QRT-PCR) and single mRNA molecule counting on the Nanostring nCounter platform. Nanostring proved to be highly sensitive, specific, and linear, with sensitivity comparable or exceeding that of RNA seq. We evaluated the prognostic significance and molecular correlates of RTK rearrangements. EGFRvIII was only detectable in tumors with focal amplification of the gene. Moreover, we found that EGFRvIII expression was not prognostic of poor outcome and that neither recurrent copy number alterations nor global changes in gene expression differentiate EGFRvIII-positive tumors from tumors with amplification of wild-type EGFR. The wide range of expression of mutant alleles and co-expression of multiple EGFR variants suggests that quantitative RNA-based clinical assays will be important for assessing the relative expression of intragenic deletions as therapeutic targets and/or candidate biomarkers. To this end, we demonstrate the performance of the Nanostring assay in RNA derived from routinely collected formalin-fixed paraffin-embedded tissue.

**Electronic supplementary material:**

The online version of this article (doi:10.1007/s00401-013-1217-3) contains supplementary material, which is available to authorized users.

## Introduction

Large-scale genomic characterization has confirmed striking heterogeneity underlying the molecular landscape of GBM and has catalogued a spectrum of tumor suppressors and oncogenes affected by deletion, amplification, mutation, and/or rearrangement. Alterations of receptor tyrosine kinases (RTKs) are especially prevalent in GBM. RTKs are a class of mitogenic signaling proteins including epidermal growth factor receptor (EGFR), platelet-derived growth factor receptor-α (PDGFRA) and MET, that are widely implicated in human oncogenesis. Indeed, high-level amplification of the *EGFR* locus represents the single most common genomic abnormality in GBM, occurring in ~45 % of all cases, and *PDGFRA* and *MET* are also frequently amplified, in 10–15 % and ~4 % of GBMs, respectively [5, 10, 31, 43]. Moreover, these amplification events have been associated with specific disease subclasses, defined by transcriptional and proteomic signatures [4, 37, 45], implying that molecular distinctions within GBM are, to some extent, mechanistically grounded in dysregulated RTK signaling.

RTK amplification in GBM is often associated with intragenic deletions and gene rearrangements, as well as extracellular domain point mutations [5, 23, 44]. As many as half of EGFR-amplified GBMs have been reported to express the variant III mutation (vIII), a 287-amino acid in-frame deletion of exons 2–7 in the EGFR extracellular domain (ECD) [42]. The resulting protein constitutively signals in a ligand-independent manner by forming homodimers or heterodimeric complexes with either wild-type EGFR or other ErbB family members [12]. EGFRvIII primarily stimulates the oncogenic PI3K/AKT pathway [17, 29], but is also known to interact with the adapter proteins Shc and Grb2, thereby activating RAS/MAPK signaling [39]. Additionally, EGFRvIII-expressing tumor cells may exert paracrine influence on their neighbors by secreting either microvesicles containing the protein itself [1] or mitogenic cytokines like IL-6 and LIF [19]. Other cancer-relevant functionalities ascribed to EGFRvIII include evasion of apoptosis [30], tumor cell invasion [22], angiogenesis [50] and stem cell self-renewal [16].

A number of additional EGFR intragenic deletions have been identified. Some, like EGFR vI (exon 1–7 deletion) and EGFR vIV (intracellular domain microdeletion), are rare [6, 9, 38, 48], while others like EGFRvII and EGFRvV are marginally more common, each accounting for more than 10 % of all GBM-associated EGFR mutations [20, 28, 32]. The vII deletion includes a small 83-amino acid stretch within the EGFR ECD [47], while EGFRvV involves a C-terminal truncation that ablates the majority of the protein’s intracellular domain, a region responsible for mediating internalization and degradation [6, 9, 48]. Functional analyses of both mutations have been complicated by their frequent co-occurrence with EGFRvIII [10]. However, recent work has demonstrated that EGFRvV is itself capable of transformation both in vitro and in mouse xenografts [7].

Intragenic rearrangements in *PDGFRA* have also been described in GBM. Similar to their counterparts in EGFR, these appear to largely occur in the context of high-level genomic amplification. An in-frame deletion in the Ig-like, extracellular domain of PDGFRA (PDGFRAΔ8,9) has been detected in up to 40 % of PDGFRA-amplified cases and results in constitutive kinase activation in vitro [21, 36]. Cases of C-terminal truncation (PDGFRAΔCt) have also been reported, although defined functional consequences remain to be established [40]. Moreover, it has yet to be determined how these mutations correlate with other oncogenic and subclass-defining molecular abnormalities in GBM.

The prevalence of RTK intragenic deletions, particularly EGFRvIII, in significant subsets of GBM has made them both attractive therapeutic targets for immunotherapeutic approaches and promising predictive biomarkers for pharmacologic receptor inhibitors [26, 35]. In this context, there remains a need to effectively detect and quantify EGFR vIII and related abnormalities in RTKs to power more detailed functional analysis and therapeutic trial stratification. Currently, most clinical labs that assess EGFRvIII status do so using non-quantitative techniques such as immunohistochemistry (IHC) and/or reverse transcription-polymerase chain reaction (RT-PCR) for the mutant transcript. Other intragenic deletions in EGFR and those of PDGFRA are not routinely measured as a component of standard patient care.

To determine the frequency and molecular context of common RTK intragenic deletions in GBM, we profiled 192 tumors from TCGA for EGFRvIII using both quantitative reverse transcriptase PCR (QRT-PCR) and a novel approach based on Nanostring nCounter technology. The latter platform was also employed to assess EGFRvII, EGFRvV, and PDGFRAΔ8,9, in the same sample set. We demonstrate that intragenic deletion mutants, particularly EGFRvIII, comprise highly variable proportions of total RTK expression in a given tumor, ranging from the majority mRNA species to only a minor component. Paired with orthogonal profiling data from TCGA, these findings now represent the most comprehensive tumor-based assessment of RTK deletion mutation in GBM to date, and provide a resource for integrated molecular analysis. Moreover, we find that Nanostring-based analysis performs robustly from formalin-fixed paraffin-embedded tissue (FFPE), thus empowering investigation and characterization of a wide dynamic range of expression of EGFRvIII and other deletion mutations in the context of clinical trials.

## Methods

### Human tissue and RNA extraction

RNA from TCGA samples was allocated from the Biospecimen Core Resource as 3 μg aliquots and sent to the MSKCC TCGA Pilot Phase Cancer Genome Characterization Center (CGCC). TCGA sample collection and RNA extraction followed published protocols [5, 44]. An additional independent tumor sample set was used to confirm the fidelity of the assay applied to FFPE, including surgical specimens collected at Memorial Sloan-Kettering Cancer Center and frozen. All patients consented prior to surgery under a protocol approved by the institution’s Institutional Review Board. Patient-matched FFPE tissue for comparison was obtained following routine processing by the Department of Pathology and diagnostic confirmation by a neuropathologist (J.T.H.). RNA was extracted from either crushed frozen tissue or 3–8 10 μm slides using the RNeasy Mini kit (Qiagen).

### Quantitative reverse transcriptase PCR

From the TCGA sample set, 275 cases with available RNA were interrogated for relative expression of wild-type EGFR and EGFRvIII by RT-PCR. 400 ng of total RNA was reverse-transcribed using the Thermoscript RT-PCR system (Invitrogen) at 52 °C for 1 h. 20 ng of resultant cDNA was used in a Q-PCR reaction using an 7500 Real-Time PCR System (Applied Biosystems) and custom-designed TaqMan gene expression Assays (EGFRvIII Forward primer: 5′CGGGCTCTGGAGGAAAAG3′; EGFRvIII reverse primer: 5′AGGCCCTTCGCACTTCTTAC3′; EGFRvIII internal primer: 5′GTGACAGATCACGGCTCGTG3′; total EGFR: pre-designed TaqMan ABI Gene expression Assays Hs01076076_m1). Primers were chosen based on their ability to span the most 3′ exon–exon junction. Amplification was carried for 40 cycles (95 °C for 15 s, 60 °C for 1 min). To calculate the efficiency of the PCR reaction, and to assess the sensitivity of each assay, we also performed a six-point standard curve (5, 1.7, 0.56, 0.19, 0.062, and 0.021 ng). Triplicates CT values were averaged, amounts of target were interpolated from the standard curves and normalized to TBP (TATA box binding protein pre-designed TaqMan ABI Gene expression Assays Hs00427620_m1). Efficiency of each reaction was determined from the standard curve of a serially diluted sample using the equation: Efficiency = 10^(−1/slope)^ − 1, where slope is fitted to CT vs. log10 (concentration). Relative quantities of TBP, EGFR and EGFRvIII were calculated from each CT[i] based on the reaction efficiencies and minimum CTs from the standard dilution curves (CT_max_) according to the formula: Quantity = (1 + Efficiency)^(CTmax−CT)^. All reactions were performed in triplicate. Samples were rejected if multiple TBP replicates failed to cross threshold in <36 cycles or if the median absolute deviation of quantified TBP across replicates was greater than 25 % (5 of 275 samples). The relative quantities of EGFR and EGFRvIII were normalized with respect to TBP.

### Nanostring

The nCounter Analysis System (Nanostring Technologies, Seattle, WA) allows for multiplexed digital mRNA profiling without amplification or generation of cDNA [13]. Briefly, mRNA is hybridized with pairs of ~50 bp probes complementary to each target. The reporter probe is tagged by a target-specific code of four fluorescent reporters at seven positions along a phage DNA backbone. The capture probe is used for immobilization on a slide and once oriented in an electric field; bound reporters are counted and annotated. A custom probe set was designed as detailed in Supplemental Table S1. Total RNA (150–300 ng) was hybridized with the codeset probes and loaded into the nCounter prep station. The samples were quantified using the nCounter Digital Analyzer.

The Nanostring platform includes negative control probes (not complementary to any endogenous mRNA) to assess background noise associated with the fluorescent barcode optical recognition system. To ensure that all samples were within the optimal range of probe density for image analysis, we confirmed that there was no systemic increase in negative control counts as a function of total number of counts recorded per sample. Raw probe counts were normalized to a panel of 8 control genes (B2M, B4GALT1, CLTC, E2F4, GAPDH, POLR2A, SDHA, and TBP) by taking the ratios of each gene’s counts per sample to the average across all samples and scaling by the median of these ratios in each sample. This normalization factor was also applied to the negative control probes counts. A detection threshold was defined for each sample as five times the mean of the negative control probe normalized counts. Of 192 samples run, three cases (TCGA-02-0021, TCGA-12-0827 and TCGA-19-1386) were excluded from analysis as outliers with low expression of the 8 control genes (possibly representing under-loading or poor hybridization).

C-terminal deletion mutation was inferred by the occurrence of relative underexpression (undercounting) of the exon 28 probe versus the exon 19 probe. The normal (wild-type) linear relationship of counts between these two probes was determined by a linear model fit to the central 90 % of the data. This model was then applied to the entire dataset to identify cases with outlier C-terminal underexpression. These cases fell into in two groups: intermediate expression of the truncation mutant (<60 % of expected c-terminal counts), or high expression (<10 %).

### RNA and DNA sequence analysis

RNA and DNA sequencing data (BAM files mapped to hg19) were obtained from TCGA through CGHub. RNA sequencing was analyzed to tabulate EGFR and PDGFRA exon junctions as described [5]. Briefly, counts were made of all EGFR and PDGFRA reads spanning exon–exon junctions and all paired exonic reads with gaps spanning one or more introns. Only reads with perfect alignment scores (CIGAR score) were considered. To account for 3′ bias in RNA sequence representation, mutant junction counts were compared with counts of normal junctions at the 3′ exon. For example, EGFRvIII expression was defined by counting reads with E1–E8 junctions and comparing to the count of reads with “wild type” E7–E8 junctions. EGFRvII was defined by E13–E16 vs. wild-type E15–E16. PDGFRA D89 was defined by E7–E10 vs. wild-type E9–E10. A junction was counted only if seen in more than one read. Exome DNA sequence data for 291 tumors were analyzed to determine read coverage within the EGFR gene in two regions: exons 2–7 (the EGFRvIII deleted region) and exons 8–22 (spanning the transmembrane and kinase domain regions). The normal ratio of counts between regions was determined by linear regression fit of the middle 90 % of ratios. This model was applied to normalize the ratios and allow accurate estimation of relative copy number of exons 2–7 vs. exons 8–22.

### DNA copy number analysis

TCGA Level 3 copy number data (normalized and segmented) were downloaded from the TGCA Data Portal for Affymetrix SNP6.0 data (Broad Institute). Copy number was inferred for exon 6 (within the 2–7 deletion) and compared with that of exon 19 (kinase domain region) to identify relative deletion. Level 2 data (normalized) for Agilent 244k aCGH data (MSKCC) were downloaded parsed into to subsets of probe values: probes residing between the midpoint of intron 1 and the endpoint of exon 7 were taken as representing the deleted region in vIII and these log2 ratios were compared to those of probes residing from the start of exon 8 through exon 21 using Student’s *t* test. A *p* value of 0.05 was taken as significant (uncorrected for multiple testing). CNA focality, a measure of how many genes are included in simple and complex aberrations, was scored for EGFR in each sample using a Genome Topography Scan method previously described (GTS [5, 43, 49]).

### Statistical analysis

Data analyses were performed in R (http://cran.r-project.org/). A prospective panel of hypotheses regarding the difference between EGFR-amplified/EGFRvIII+ and EGFR-amplified/EGFRvIII− were evaluated by Fisher’s exact test for discrete events and by a two-sided student’s *t* test for continuous variables and *p* values were adjusted by FDR. In all cases, the EGFRvIII-high and low positives (EGFRvIII in >1 % of EGFR transcripts), along with EGFR-high positives alone (EGFRvIII in >10 % of EGFR transcripts), were independently compared with wild-type EGFR-amplified tumors. Exploratory searches for differentially expressed genes and miRNAs were performed using empirical Bayes analysis within the Linear Models for Microarray Analysis package implemented in R [41].

## Results

### QRT-PCR and Nanostring profiling reveal a wide range of EGFRvIII expression in EGFR-amplified GBM

**Fig. 1:**
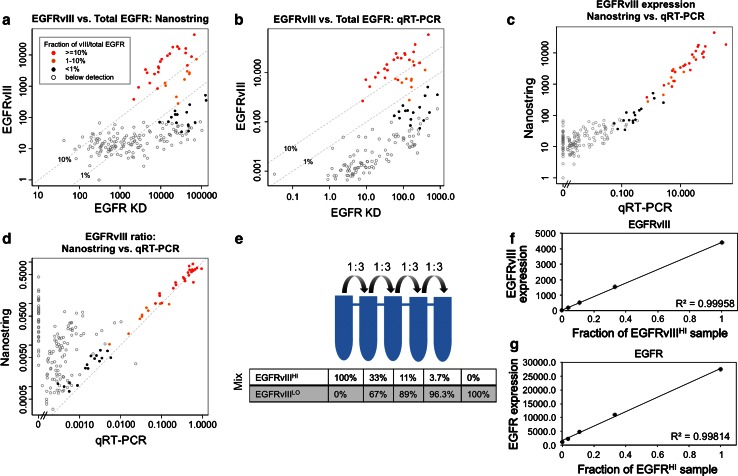
Expression of EGFRvIII as a fraction of total EGFR is quantified by Nanostring assay and qRT-PCR in 189 GBMs. **a** Expression of EGFRvIII (exon 1–8 junctional probe) is shown as a function of EGFR kinase domain (*KD*), determined by normalized Nanostring (*NS*) counts. Expression levels are classified as high [*red* mutation in >10 % transcribed allelic fraction (*TAF*)], intermediate (*orange* 1–10 % TAF), marginal (*black* <1 % TAF) or negative (*open circles*). These color assignments are carried through panels **b**–**d**. **b** Correlation of EGFRvIII expression between NS and qRT-PCR. Normalized expression levels are plotted for EGFRvIII and KD from the Taqman assay (see “Methods”). Samples are colored according to NS expression classification from Fig. 1a. **c** Cross-platform correlation of EGFRvIII epression, NS vs. qRT-PCR. **d** Cross-platform correlation of EGFRvIII as a fraction of total EGFR, NS vs. qRT-PCR. **e** Experimental design of dilution experiment to establish linearity of the Nanostring assay. A sample with high relative expression of EGFRvIII was diluted with a sample negative for EGFRvIII expression, maintaining a constant 250 ng of total RNA in each reaction. **f** Counts of EGFRvIII and EGFR KD as a function of diluted fraction of EGFRvIII-containing sample

To assess the frequency and extent of EGFRvIII mutations in GBM, we developed two independent methods for quantitative measurement of EGFRvIII. After initial validation, these assays were applied to mRNA extracted from GBM samples as part of the initial GBM TCGA Pilot Project [5, 44]. Specifically, a TaqMan-based qRT-PCR approach was compared to a Nanostring nCounter assay (NS), each targeting both the exon 1–8 junctional region of EGFRvIII (E1–8) and the EGFR kinase domain (KD) as well as select control genes (see “Methods”). After normalization, EGFRvIII expression (E1–8) was compared to total EGFR-encoding mRNA (KD). The expression of EGFRvIII was categorized using NS as absent (<fivefold above mean negative control counts, see “Methods”) or present as a fraction of overall EGFR: <1 % (black), 1–10 % (orange) or ≥10 % (red). As shown in Fig. 1a, b, both platforms demonstrated a similar pattern of expression of EGFR overall (KD) and of the vIII variant ranging over three orders of magnitude. EGFRvIII measures were well correlated across platforms (Fig. 1c). Notably, this correlation was seen even among cases with NS counts below the negative detection threshold (open circles, Fig. 1c) suggesting either detection of very low levels of EGFRvIII expression or a component of non-specific hybridization common to both platforms. The estimated ratio of EGFRvIII to total EGFR was highly concordant between platforms (Fig. 1d). Linearity of the Nanostring readout was confirmed by performing serial dilution of an EGFR vIII-high positive sample into an EGFR vIII-negative sample, yielding near-perfect correlation (*R* ^2^ >0.99) (Fig. 1e–g).

### Validation of the Nanostring assay with RNA-seq

**Fig. 2:**
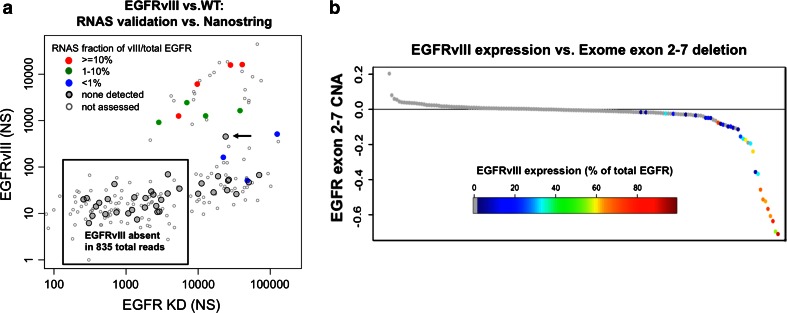
Comparison with orthogonal platforms **a** EGFRvIII vs. total EGFR as determined by Nanostring is plotted. EGFRvIII expression was determined independently from TCGA RNA-seq analysis (RNAS). *Red* denotes cases with >10 % TAF by RNAS, *green* 1–10 % and *blue* <1 %. *Black circles* filled with *gray* mark cases where no RNAS reads identified EGFRvIII; *empty circles* mark cases for which RNA-seq data were unavailable. **b** EGFRvIII expression was compared with genomic loss of EGFR exons 2–7 in 157 cases for which both RNA and DNA (exome) sequencing data were available. Samples are ordered by the magnitude of exon 2–7 deletion inferred from DNA seq coverage. Expression was determined by the ratio of VIII junction RPKM to total EGFR

To more definitively ascertain the performance of the Nanostring assay, we correlated our findings with transcriptome sequencing (RNA-seq) data from TCGA, available for 61 samples in our study set (47 with NS data). We found near universal agreement between “positive” status on the Nanostring platform and the presence of bridging RNA-seq reads spanning the exon 1–8 breakpoint in EGFRvIII (Fig. 2a, colored dots). Only one discordant sample, judged positive by Nanostring but lacking confirmed junctional reads by RNA-seq, was identified (Fig. 2a, arrow). However, this absence of reads is within sampling error based on the RNA-seq coverage (225×) given transcribed allelic fraction of vIII at 1.6 % as estimated by NS. RT-PCR confirmed that this discrepant case was EGFRvIII+, with a low transcribed allelic fraction (~9 %). Overall, the coverage of counts by NS was much higher than reads by RNA-seq. Among the 47 samples with both RNA sequencing and NS data, the mean coverage of EGFR by RNA-seq was 450× compared to a mean of 12,000 counts in the NS data. Overall, 60 % of samples, comprised largely of tumors without high-level EGFR amplification, did not reach 100× coverage at the EGFR locus by RNA-seq, precluding the definitive detection of a minor (<1 %) transcript population.

To estimate a lower bound for EGFRvIII expression among samples with relatively low EGFR expression, we pooled all RNA-seq reads from 25 cases with NS counts for EGFR <10,000 and EGFRvIII counts up to 100 (box, Fig. 2a). Among a total of 853 reads of EGFR, the vIII junction was not seen once, suggesting that any expression of EGFRvIII in this population is <1 % at most (95 % confidence interval is 0–0.43 %).

Because EGFRvIII mutation is associated with genomic deletion of exons 2–7, we sought to determine whether assessment of this deletion by DNA copy number data could serve as a surrogate for RNA-based determination. TCGA exome sequencing data for 291 GBMs were analyzed to determine the read coverage within the EGFR exon 2–7 interval and a control interval from exon 8–22 (see “Methods”). EGFRvIII expression was then compared for 157 cases for which RNA-seq data were also available. As shown in Fig. 2b, all samples that expressed EGFRvIII mRNA showed evidence of a relative loss of exons 2–7 at the DNA level, and samples with the greatest copy number change showed the highest vIII expression levels. Exon 2–7 deletion inferred from exome coverage was able to predict vIII expression with at least 80 % sensitivity at 95 % specificity (Supplemental Fig. S1a). In the TCGA dataset, exome coverage was a more sensitive detector of intragenic deletion than microarray data, specifically the Affymetrix SNP6.0 and Agilent array-CGH platforms, although all DNA measures lacked the sensitivity of mRNA assays (Supplemental Fig. S1b, c).

### Nanostring profiling effectively detects EGFRvII, EGFRvV, and PDGFRAΔ8,9 in small subsets of RTK-amplified GBM

Using analogous approaches to that employed for EGFR vIII, we developed Nanostring assays for the detection of EGFR vII and PDGFRAΔ8,9 based on their specific breakpoint regions. Additionally, we sought to measure EGFR vV transcript by including a probe set in our Nanostring panel directed against the C-terminal of EGFR (EGFR C-term), allowing detection based on the count ratio of the C-term and kinase domains.

**Fig. 3:**
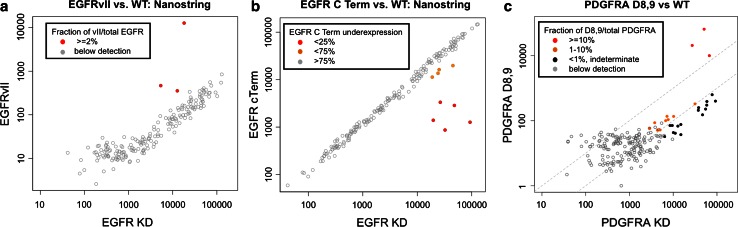
Assessment of EGFRvII, EGFRvV and PDGFRAΔ8,9 using Nanostring probes. **a** Probes targeting the aberrant junctions characterizing EGFRvII expression levels are classified as positive (*red* mutation in >2 % TAF), or not detected (*open circles*). **b** EGFRvV (C-terminal deletion) is detected by relative under-representation of exon 28 vs. exon 19 harboring the kinase domain (*KD*). **c** PDGFRAΔ8,9 expression is stratified as in Fig. 1a

Applying these assays to the TCGA cohort revealed distinct clusters of outliers characterized by high-level expression of mutant transcript (Fig. 3a–c). For EGFRvII, we detected three samples expressing the mutant allele over a threshold of 2 % of total EGFR counts (and with EGFRvII count >5× negative controls). RNA-seq data were available for two of the three cases and confirmed expression of the vII junction in both (Supplemental Fig. S2). Although NS data demonstrated a strong correlation between total EGFR expression and a low level (<1 %) of EGFRvII counts, RNA-seq failed to confirm the vII junction in most of these cases (Supplemental Fig. S2).

For EGFR vV, we stratified positive samples into “high” and “low” on the basis of percent composition of C-terminal deleted transcript (Fig. 3b, see “Methods”). Five cases, accounting for 2.6 % of all tumors and 6 % of the EGFR-amplified subset, exhibited marked C-terminal loss (>90 % EGFR mutated; Fig. 3b, red). Interestingly, a recent TCGA report examining genomic alterations in EGFR by microarray-based copy number analysis demonstrated that these same five samples exhibit profoundly reduced levels of the EGFR C-terminal exon [7]. Moreover, our data also indicated lower expression levels of the C-terminal deletion transcript in four previously unidentified samples (Fig. 3b, orange). Taken together, 4.7 % of cases overall (10.8 % of EGFR-amplified cases) showed evidence of C-terminal truncation in a significant proportion of EGFR transcript. In addition to truncations of the C-terminus, deletion mutations affecting exons 25–27 have been identified by analysis of RNA sequencing data in the TCGA dataset [5]. These intragenic deletions do not include the terminal exon and therefore would not be detected by the NS panel used in this study.

High-level PDGFRAΔ8,9 expression was identified in three samples, representing 1.6 % of all tumors and 17.6 % of the PDGFRA-amplified subset (Fig. 3b). None of the high-expressing cases had RNA-seq data available. However, analysis of the remaining cases identified that a low level of the Δ8,9 junction could occasionally be detected as a minor fraction of expressed PDGFRA [5]. The complete dataset of Nanostring, QRT-PCR and RNA-seq results is available at the public portal (https://tcga-data.nci.nih.gov/docs/publications/gbm_2013/).

### Analysis of paired specimens suggests robust performance of Nanostring-based intragenic deletion profiling in FFPE specimens

**Fig. 4:**
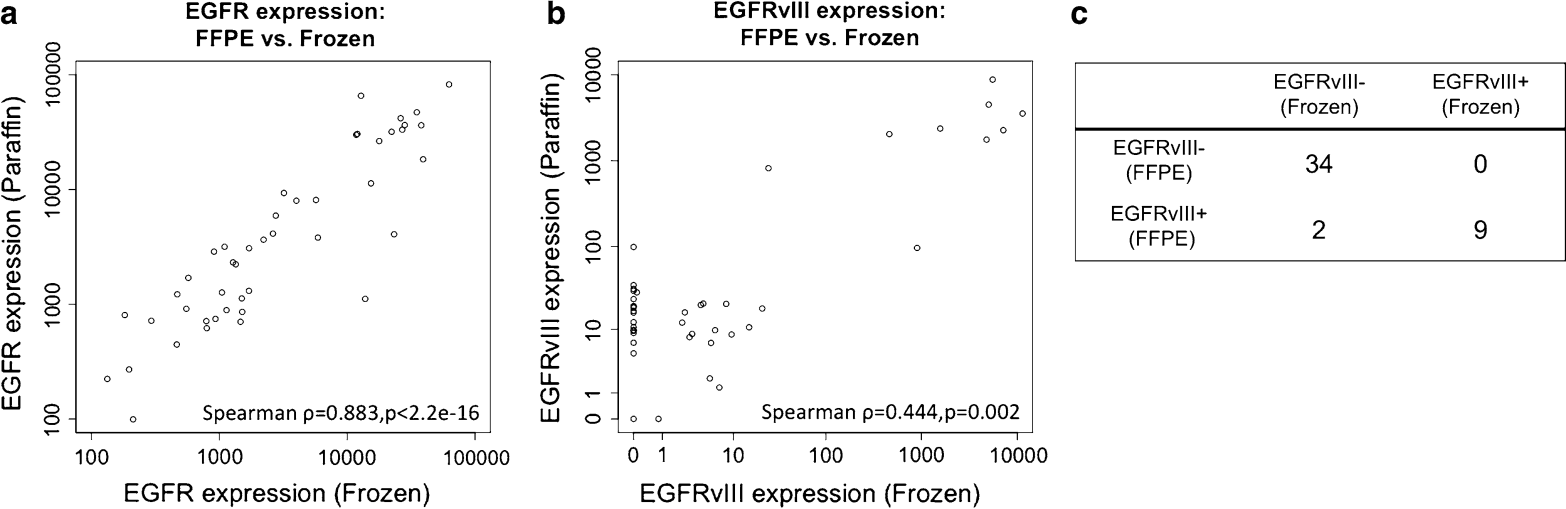
Performance of Nanostring assay applied to suboptimal material. Counts of EGFR-WT (**a**) and EGFRvIII (**b**) are correlated between patient-matched samples maintained by optimal, flash-frozen, and suboptimal, versus formalin-fixed paraffin-embedded samples (*FFPE*), preservation methods. **c** Concordance of NS assay as a binary classifier from FFPE and frozen material

FFPE tissue blocks remain the standard for clinical sample processing in medical centers, despite suboptimal preservation of biomaterials like nucleic acids. To assess the performance of our Nanostring assay in FFPE samples, we utilized an independent cohort of patient-matched fresh-frozen and FFPE specimens (*N* = 45). Total RNA extracted from FFPE and fresh-frozen samples was analyzed for RTK intragenic deletions in both EGFR and PDGFRA. We found strong correlations between FFPE and fresh-frozen RNA in the levels of total EGFR (Spearman rho = 0.883, *p* < 2.2e-16, Fig. 4a) and EGFRvIII (Spearman rho = 0.444, *p* = 0.002, Fig. 4b). For EGFRvIII and other deletion mutations, the majority of cases expressed levels below background. Consequently, the variance of noise at near-zero counts reduced correlations for the population as a whole. The NS assay also appeared to perform well in the context of presumptive clinical decision-making. Specifically, a binary classifier for EGFRvIII (negative/borderline versus positive) applied to results from FFPE material demonstrated 100 % sensitivity and 94 % specificity (Fig. 4c). Moreover, measured EGFRvIII counts in the two identified “false positives” were in the low-positive range, indicating superior performance in samples containing high levels of EGFRvIII transcript. As confirmation of the specificity of the Nanostring assay for EGFRvIII in gliomas, no positive results were observed in RNAs from 269 non-glioma samples (not from TCGA) including 97 lung adenocarcinomas, 23 ductal breast carcinomas, 36 colon carcinomas, 21 thyroid carcinomas, 25 osteosarcomas, 12 chondrosarcomas, 18 cholangiocarcinomas, and 37 samples of non-neoplastic lung tissue (Y. Suehara, M. Ladanyi, unpublished data). Concordance between FFPE and frozen was comparable for the other deletion mutation probes (Supplemental Fig. S3).

**Fig. 5:**
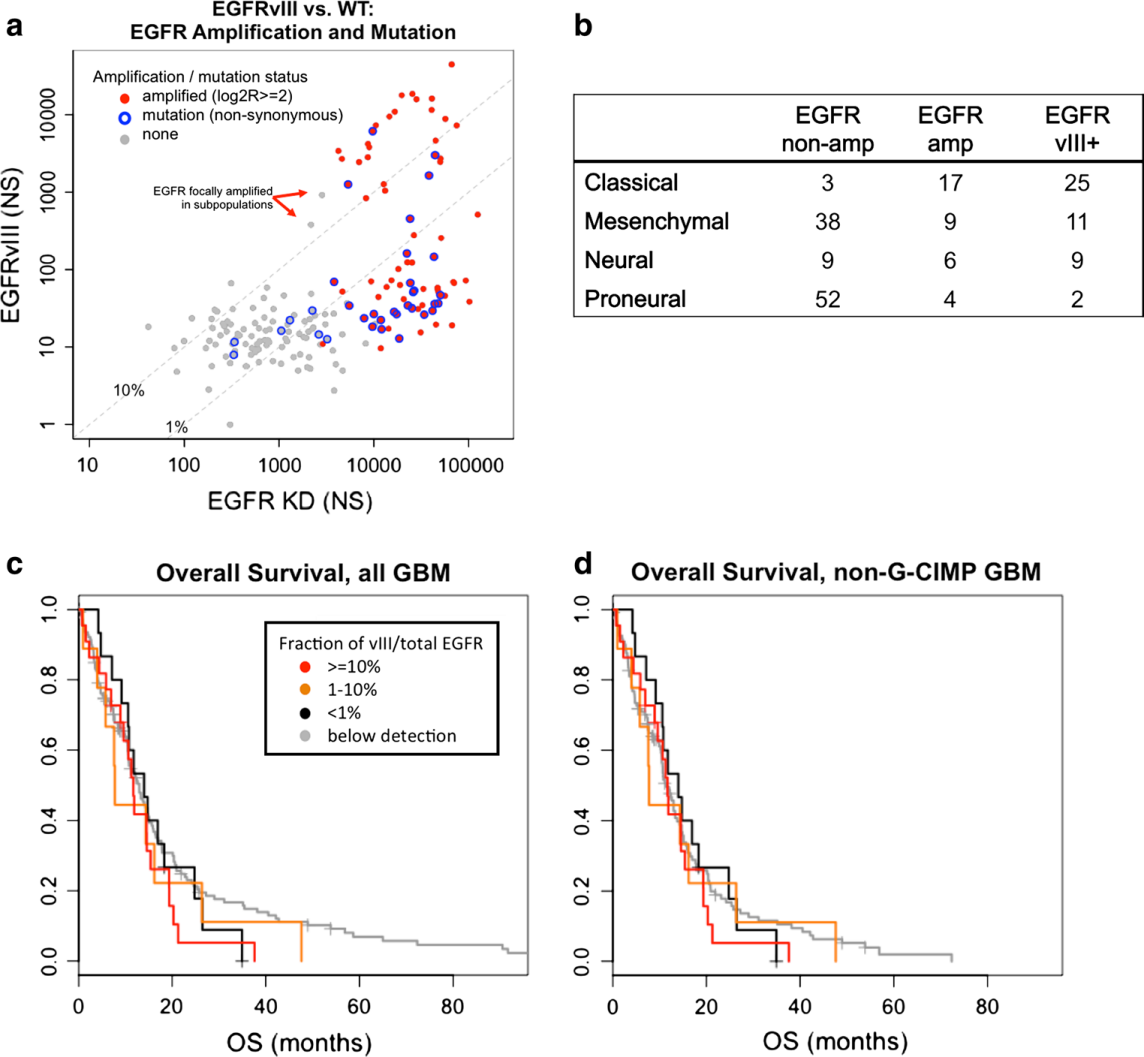
Genomic and clinical correlates of EGFRvIII expression. **a** Significant EGFRvIII expression is exclusively found in tumors with amplification of EGFR. NS counts of EGFRvIII expression are plotted with respect to kinase domain counts. *Blue circles* denote samples with EGFR point mutation. *Red* denotes tumors with high-level amplification of the EGFR locus (aCGH log2 ratio >2). For two samples with high EGFRvIII expression, but log2 ratios below 2 (*red arrows*), aCGH demonstrates focal CNA in a pattern consistent with high-level gene amplification in a subpopulation of cells (and demonstrated by FISH for one of the two cases [43]). **b** Association between EGFR status and transcriptomal subclass. **c** Overall survival of patients stratified by EGFRvIII status. **d** Overall survival of patients stratified by EGFRvIII status excluding G-CIMP tumors, which are known to have a more favorable prognosis

Consistent with extensive prior literature [12, 42, 51], we found a tight association between EGFRvIII mutation and high-level EGFR amplification in our sample set defined by aCGH log2 ratio >2 (Fig. 5a). Only two cases with high-level EGFRvIII expression demonstrated log2 ratios below 2 (TCGA-06-0156 and TCGA-08-0360). However, examination of aCGH data for both cases revealed focal CNA of the EGFR locus in a pattern consistent with high-level amplification within a subpopulation of cells, as confirmed by FISH for one sample (TCGA-06-0156) [43]. We confirmed this observation by evaluating all 116 cases with both RNA-seq and aCGH data available (Supplemental Fig. S4). EGFRvIII transcript was detected by sequencing in 38 of the 64 cases with focal EGFR amplification (59 %). No EGFRvIII transcript was found among the subset of 52 unamplified cases, while the WT junction was read a total of 1,789 times (95 % CI for EGFRvIII 0–0.21 %). These results establish that GBMs rarely if ever express high levels of EGFRvIII in the absence of focal amplification of the locus. Additionally, there is no evidence of promiscuous low-level expression that one might expect if EGFRvIII were the result of common splicing variation.

### EGFRvIII does not independently correlate with specific molecular and/or clinical features within EGFR-amplified GBM

EGFR amplification and EGFRvIII expression were both associated with the classical transcriptional subclass (Fig. 5b). However, this association was not independently significant for EGFRvIII. Moreover, EGFRvIII positivity at any level was not predictive for overall survival in GBM (Fig. 5c). An apparent overall difference of long-term survivors disappears after excluding patients with the distinct phenotype of GBM CpG island hypermethylation (G-CIMP [34]) (Fig. 5d). Cox proportional hazards regression models fit either EGFRvIII counts, EGFR-WT counts, or EGFRvIII/EGFR ratio revealed no significant prognostic value for any of these parameters.

In a further effort to identify molecular features distinguishing EGFRvIII-mutant tumors from their wild-type EGFR-amplified counterparts, we utilized copy number, gene expression, and histopathological data for our TCGA sample set [5]. We first prospectively tested a limited set of selected molecular and histopathological parameters including small cell histology or pseudopalisading necrosis; deletion/mutation of *TP53*, *NF1*, *PTEN*, *CDKN2A*, *CDKN2C*, and *RB1*; amplification of CDK4/6; mRNA expression of IL6 or LIF, MMP13 and BCL-XL. This demonstrated no statistically significant differences between EGFRvIII^HI^ (*n* = 20) and EGFRvIII-negative tumors (*n* = 37) within the EGFR-amplified subset (Supplemental Table S2). We then tested all TCGA-measured variables using empirical Bayesian analysis and found no specific copy number events or mRNAs, miRNAs, or proteins whose differential expression between EGFRvIII-positive, and wild-type EGFR-amplified tumors reached statistical significance. Similarly, no scored histopathological features were found to delineate mutant and wild-type samples by Chi-squared analysis.

### Molecular and clinical features of GBMs harboring other RTK intragenic deletions

**Fig. 6:**
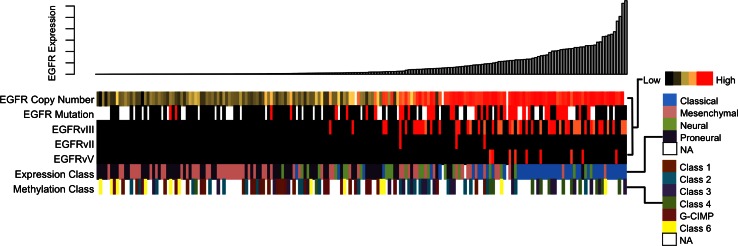
Molecular context of EGFR alterations in GBM. From *top to bottom* EGFR mRNA expression, DNA copy number, deletion mutation expression, transcriptomal and methylation subclass are reported for each sample

We screened other available molecular data to identify features that might be correlated with expression of EGFRvII, EGFRvV, and PDGFRAΔ8,9. As expected, PDGFRAΔ8,9 was seen at high levels only in the context of high-level PDGFRA amplification and its presence exclusively within the proneural expression subclass (Fig. 6; Supplemental Fig. S5c). Similarly, both EGFRvII and EGFRvV-positive tumors were invariably amplified for EGFR (Fig. 6). Somewhat surprisingly, we found that the three EGFRvII-expressing tumors were all assigned to the mesenchymal expression subgroup (Supplemental Fig. S5a). By contrast, EGFR vV-positive tumors, particularly those exhibiting strong positivity, were primarily designated as classical, although mesenchymal and neural classifications were also seen, primarily for lower expressers (Supplemental Fig. S5b).

Detailed analysis of gene expression data revealed that, unlike for EGFRvIII, EGFRvII positivity correlated with a defined gene signature relative to wild-type EGFR-amplified tumors. In total, we observed 27 genes with statistically significant upregulation in EGFRvII tumors (Supplemental Table S3). Of these, 26/27 genes were similarly upregulated in mesenchymal tumors versus all others with two genes also upregulated in the setting of EGFR amplification, likely reflecting the transcriptional and genomic features of the three EGFRvII-positive tumors. Gene ontology (GO) analysis revealed significant correlations between the EGFRvII gene signature and chemokine activity, signal transduction, cellular locomotion (Supplemental Table S4). We did not identify genes or expression signatures similarly associated with either EGFR vV or PDGFRAΔ8,9 positivity relative to wild-type receptor-amplified tumors, nor were specific miRNAs or copy number events correlated with any of the three deletion mutants. Moreover, consistent with earlier work demonstrating the frequent co-occurrence of EGFR vII and vV with vIII [10], we found that all EGFR vII-positive tumors and 44 % (4/9) of EGFR vV-positive tumors in our sample set also expressed high levels of EGFR vIII (Fig. 6). Finally, overall survival was not significantly different between either EGFR vII or vV-mutant tumors and all others (Supplemental Fig. S6a-S6b). And while Kaplan–Meier analysis identified poorer outcomes for PDGFRAΔ8,9 tumors (*p* = 0.0257), sample size limits the robustness of this finding (Supplemental Fig. S6c).

## Discussion

The Cancer Genome Atlas GBM initiative has recently completed analysis of a molecularly and clinically annotated dataset of unprecedented detail for over 500 tumors [5]. This project was initiated in 2006, before the advent of high-throughput DNA and RNA sequencing technologies. As a result, the initial TCGA marker paper in 2008 had no direct measure of intragenic deletion mutations despite these being the most common forms of RTK activation in GBM [44]. Our study aims to provide this annotation for 189 TCGA tumors, quantified by Nanostring and verified for EGFRvIII quantitatively by RT-PCR. As technology has advanced, TCGA has subsequently performed RNA sequencing for 164 of the most recent cases, 47 overlapping our NS dataset. Together, the NS and RNA-seq data provide a quantitative annotation of common RTK deletion variants for 306 tumors. The ability to cross-reference expression levels of EGFRvIII and other RTK deletions against the clinical and detailed molecular data in TCGA provides a valuable resource to better understand the molecular context in which these mutations are found.

We found no prognostic significance of EGFRvIII expression in the primary GBMs comprising TCGAs dataset. This is consistent with some prior studies performed on independent datasets [2, 15, 24]. Our global analysis of molecular correlates of EGFRvIII and other deletion mutations revealed that, for the most part, tumors with these mutations were also not distinguished by specific molecular features compared to their wild-type RTK-amplified counterparts. This analysis does not imply that EGFRvIII expression has no molecular effects, but rather that detecting these effects in the TCGA data will require prospective testing of select hypotheses. The TCGA dataset also does not reflect differences in subcellular localization, post-translational modification, or degradation of EGFR protein, any or all of which might be impacted distinctly by vIII mutation [7, 14, 25]. Nonetheless, the global similarity of EGFR-amplified tumors, whether EGFRvIII positive or negative, suggests that common features are shared by GBMs with EGFR activation by any means, and that neomorphic functions specific to EGFRvIII may not be strongly influential on the tumor phenotypes measured here. In contrast, EGFRvII-expressing GBMs do appear to have an expression signature distinct from most other EGFR-amplified tumors. It is likely that this finding reflects the association of vII mutation with mesenchymal rather than classical transcriptional subclass, as 26/27 EGFRvII signature genes (96 %) were also associated with non-vII-expressing mesenchymal GBMs in the same analysis.

Because RTK mutations are typically associated with gene amplification in GBM, there can be a wide range of expression of mutant and wild-type alleles [10], and these levels may vary tumor-to-tumor and even cell-to-cell [19, 33]. Earlier work has shown that multiple mutations can affect a single EGFR allele [10]. Recent analysis of TCGA RNA-seq data revealed that multiple EGFR deletion and point mutations were often expressed in the same tumor at different allelic frequencies [5]. We observed a high rate of co-occurrence between different EGFR deletion mutants in our sample set—100 % of EGFRvII and 44 % of EGFRvV-positive tumors also harbored EGFRvIII. The biological significance of multiple coincident EGFR deletion mutations in the same tumor remains unclear. Interestingly, some evidence supports the possibility of functional heterodimerization involving mutant and wild-type receptors, which may play a driving role in the maintenance of EGFRvIII as a minority species in a transformed cell [11, 25].

In addition to providing a molecular annotation resource, this report describes a transcript-based quantitative assessment of EGFRvIII, along with other deletion mutants operative from a relatively small amount of biomaterial. Our Nanostring-based assay exhibited notable linearity even at low levels of transcript expression and performed well in the context of FFPE starting material. This latter finding, consistent with a number of prior studies, likely reflects the absence of PCR in the Nanostring workflow. Indeed, such signal amplification can accentuate systematic error in quantitative measurements, particularly in the context of compromised starting material. Methods for the routine detection of RTK deletion mutants like EGFRvIII from surgical biopsy material remain poorly standardized and non-quantitative. Immunohistochemistry and/or RT-PCR are the predominant assays used in the clinical setting, with results typically interpreted in a binary fashion as either “positive” or “negative”. While such readouts are practical for certain applications and are currently less expensive, they do not accurately capture the molecular and cellular heterogeneity known to characterize GBM, nor are they readily quantifiable. Moreover, recent analysis has shown that multiple EGFR point and deletion mutations can be expressed in the same tumor at different allelic frequencies [5].

Overall, our findings agree with prior literature, both in the proportion of cases where Nanostring was suggestive of EGFRvIII—24 % of total and 54 % of EGFR-amplified—as well as the proportion of high-level expressers (10.6 % overall) [3, 51]. These figures include cases in which EGFRvIII was detected in <1 % of EGFR transcripts, where the biological significance and contribution of technical noise is unknown. Available RNA-seq data from overlapping TCGA samples provided strong cross-validation, as detectable reads for EGFRvIII were present in all but one of the samples designated >1 % by Nanostring. Similar correlations were observed for EGFRvII, EGFRvV, and PDGFRA Δ8,9, albeit on fewer samples. The higher sensitivity of the Nanostring assay to detect mutant transcripts at low expression levels may be related to better coverage depth. In all cases, Nanostring provided markedly higher read counts than RNA-seq (typically 50- to 100-fold greater). Next-generation sequencing costs can only be expected to fall in the coming years, enabling higher read counts routinely. Nevertheless, the limited tissue specimens available in the clinical setting may be insufficient to supply the microgram quantities of RNA typically required for transcriptome sequencing, and a significant proportion of clinical material is FFPE. Thus, assay platforms that are both cost- and resource-effective will continue play central roles in clinical management. Additionally, the ability of the Nanostring nCounter to assess up to 800 mRNAs simultaneously, while not comprehensive, should allow the multiplexing of RTK deletion mutants with a number of other transcripts and gene expression signatures of interest without increasing the required biomaterial.

RTK deletion mutants, along with their wild-type receptors, remain therapeutic targets of considerable potential for GBM. The lack of encouraging clinical results with RTK inhibition thus far may reflect, in part, inadequate drug penetration, lack of molecular stratification in clinical trials and signaling feedback mechanisms [8, 18, 27]. Cellular and molecular heterogeneity involving wild-type and mutant RTK composition, as we observed in this study, likely complicates strategies to effectively inhibit oncogenic signaling. Indeed, investigations carried out in vitro and in human patients indicate that the inhibitor sensitivity profiles of wild-type EGFR and EGFRvIII are distinct [46]. In this respect, our findings and those of others support the notion that a successful therapeutic strategy will require the effective inhibition of both mutant and wild-type receptor at concentrations achievable in the target tissue. Indeed, incomplete targeting of EGFR isoforms could simply drive tumor evolution toward a cellular population expressing an untargeted (resistant) variant. For loci that are commonly amplified in GBM, “quantitative genotyping” of the amplicons and their contained mutations may be a requirement to unambiguously establish their value as predictive and prognostic markers, particularly if established pathogenic mutations exist as minority species. Consequently, methodologies such as those described in this report may prove vitally important to standard clinical practice.

## Electronic supplementary material

Below is the link to the electronic supplementary material.

Supplemental Figure S1: Genomic deletion of exons 2–7 predicts EGFRvIII expression with variable sensitivity. Receiver Operating Characteristic (ROC) curves were calculated for the prediction of EGFRvIII mRNA expression as a function of the difference in DNA copy number measured for exons 2–7 vs. exons 8–22. Panels compare different copy number measurement methods: (a) exome sequence counts (b) array-CGH (Agilent) and (c) SNP array (SNP6.0, Affymetrix) Exome sequencing was highly sensitive, detecting > 80 % of EGFRvIII-mutant cases at 10 % FDR and 100 % at 20 % FDR. Both microarray platforms showed < 60 % sensitivity at 10 % FDR (TIFF 6079 kb)

Supplemental Figure S2: RNA-seq validation of EGFRvII is plotted over the distribution of Nanostring counts. Two samples with intermediate expression were concordant across methods (1–10 %). RNA data was unavailable for the single high-expressing case. Red denotes cases with > 10 % TAF, green 1–10 % and blue < 1 %. Black circles filled with gray denote cases where no reads identified EGFRvII. Empty circles mark cases for which RNA-seq data was unavailable (TIFF 2327 kb)

Supplemental Figure S3: Comparison of Nanostring performance between patient-matched fresh-frozen versus formalin-fixed paraffin-embedded samples (FFPE) for (a) EGFRvII, (b) EGFR CTerm, (c) PDGFRA, and (d) PDGFRAD8,9. Indicated in each plot is the Spearman correlation coefficient and the test statistic of the Pearson’s product moment correlation coefficient (TIFF 7931 kb)

Supplemental Figure S4: EGFRvIII expression, determined from RNA sequencing (RNAS), is restricted to GBMs with focal EGFR amplification. The amplitude and focality of EGFR locus copy number alterations are plotted for cases for which RNA-seq and array-CGH data were available. Focality of CNA is determined based on the extent of Chr7 with log2 ratio at-or-below that of EGFR, using the GTS algorithm previously described [5, 49]. Samples with focal CNA are red (focality < 5mb). Blue denotes cases where EGFRvIII was detected in more than a single read. TCGA-06-0156 was confirmed to harbor high-level EGFR amplification in a subpopulation of tumor cells by FISH [43]. EGFRvIII expression was not found among 52 samples without focal CNA (black dots), while the wild-type junction was read a total of 1789 times. (TIFF 1783 kb)

Supplemental Figure S5: Association of transcriptional subclass and (a) EGFRvII, (b) EGFRvV, and (c) PDGFRAD8,9. (TIFF 2774 kb)

Supplemental Figure S6: Association of overall survival and (a) EGFRvII, (b) EGFRvV, and (c) PDGFRAD8,9. (TIFF 6064 kb)

Supplemental Table S1: Sequences of probes used for Nanostring nCounter Assay. Table S2: List of variables for prospective analysis contrasting EGFRvIII + and EGFR-WT-amplified tumors. Table S3: Genes differentially expressed between EGFRvII + and EGFR-WT amplified tumors. Table S4: GO analysis of EGFRvII-associated genes. (XLSX 46 kb)
