# Supplementary figures and images for: Quantitative assessment of intragenic receptor tyrosine kinase deletions in primary glioblastomas: their prevalence and molecular correlates
Source: Acta Neuropathol. 2013 Nov 29;127(5):747–59. doi: 10.1007/s00401-013-1217-3 (PMC3984672; doi:10.1007/s00401-013-1217-3)

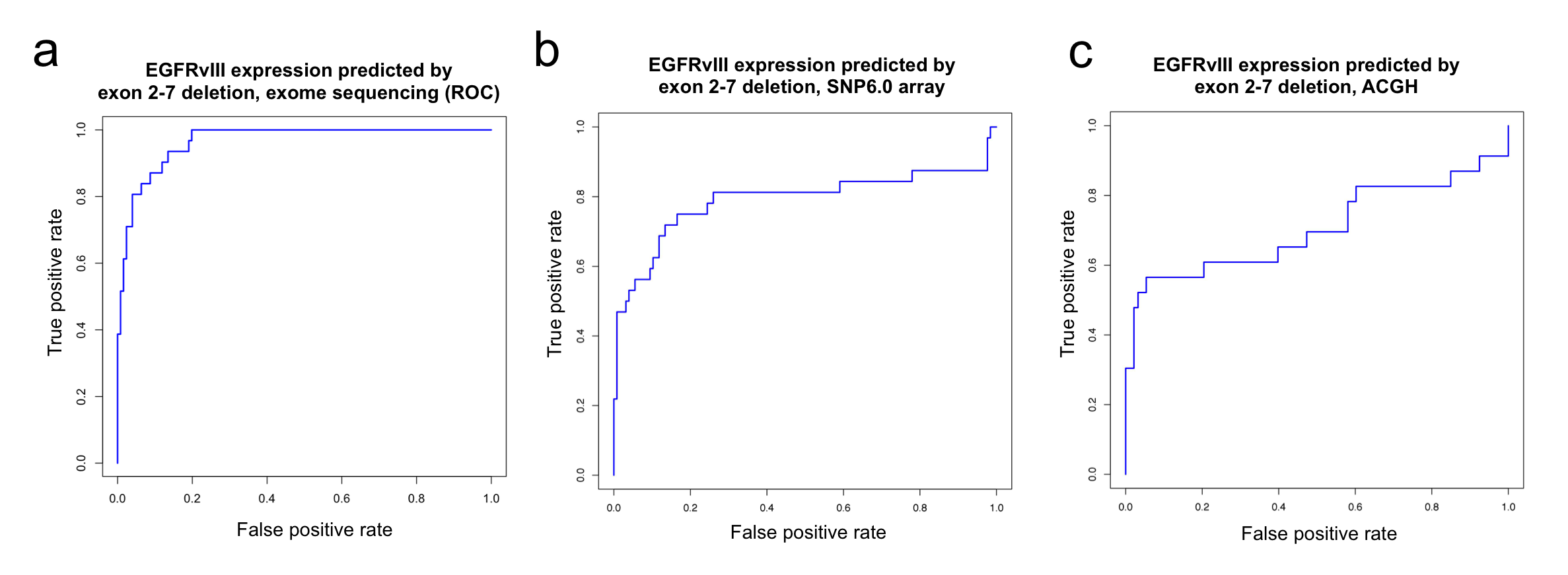

Supplement: Supplementary file 1 — Supplemental Figure S1: Genomic deletion of exons 2–7 predicts EGFRvIII expression with variable sensitivity. Receiver Operating Characteristic (ROC) curves were calculated for the prediction of EGFRvIII mRNA expression as a function of the difference in DNA copy number measured for exons 2–7 vs. exons 8–22. Panels compare different copy number measurement methods: (a) exome sequence counts (b) array-CGH (Agilent) and (c) SNP array (SNP6.0, Affymetrix) Exome sequencing was highly sensitive, detecting > 80 % of EGFRvIII-mutant cases at 10 % FDR and 100 % at 20 % FDR. Both microarray platforms showed < 60 % sensitivity at 10 % FDR (TIFF 6079 kb) [file 401_2013_1217_MOESM1_ESM.tif]

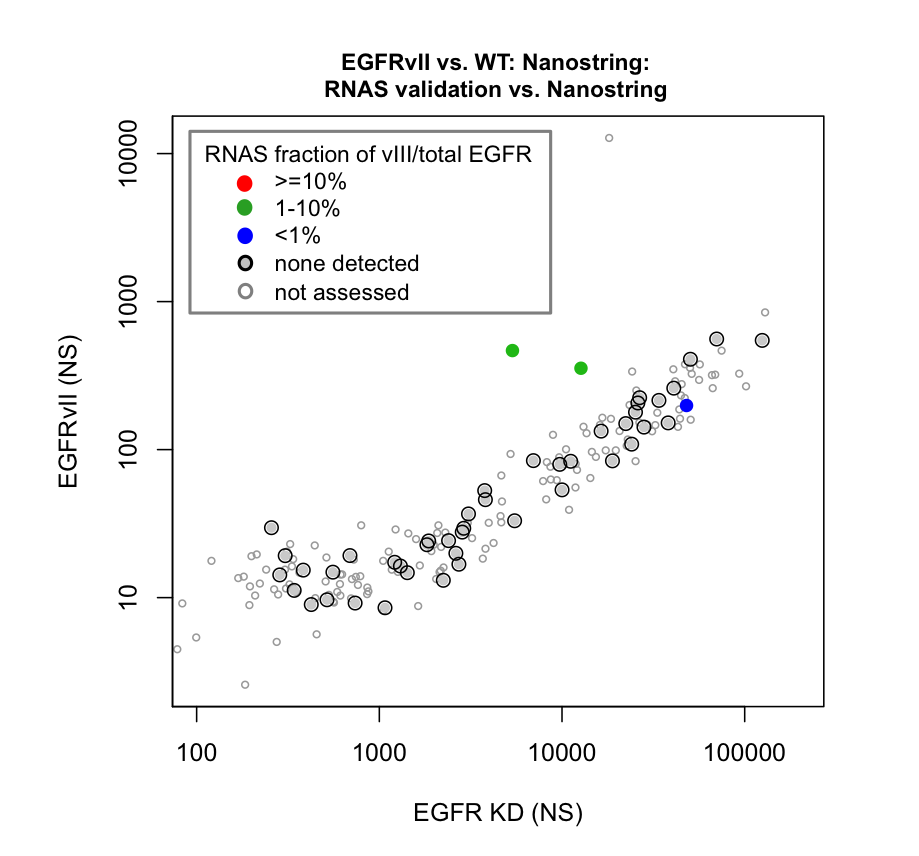

Supplement: Supplementary file 2 — Supplemental Figure S2: RNA-seq validation of EGFRvII is plotted over the distribution of Nanostring counts. Two samples with intermediate expression were concordant across methods (1–10 %). RNA data was unavailable for the single high-expressing case. Red denotes cases with > 10 % TAF, green 1–10 % and blue < 1 %. Black circles filled with gray denote cases where no reads identified EGFRvII. Empty circles mark cases for which RNA-seq data was unavailable (TIFF 2327 kb) [file 401_2013_1217_MOESM2_ESM.tif]

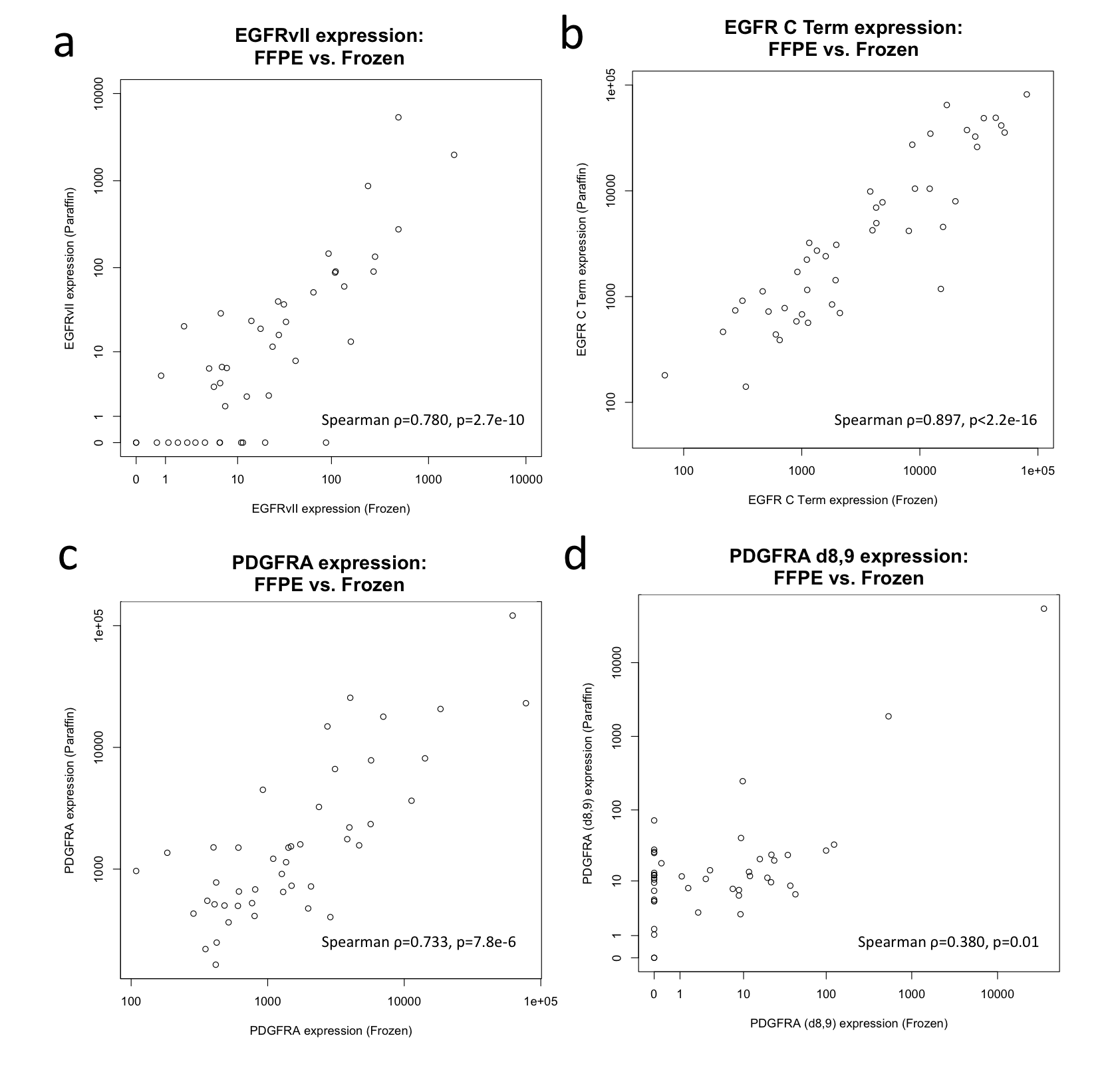

Supplement: Supplementary file 3 — Supplemental Figure S3: Comparison of Nanostring performance between patient-matched fresh-frozen versus formalin-fixed paraffin-embedded samples (FFPE) for (a) EGFRvII, (b) EGFR CTerm, (c) PDGFRA, and (d) PDGFRAD8,9. Indicated in each plot is the Spearman correlation coefficient and the test statistic of the Pearson’s product moment correlation coefficient (TIFF 7931 kb) [file 401_2013_1217_MOESM3_ESM.tif]

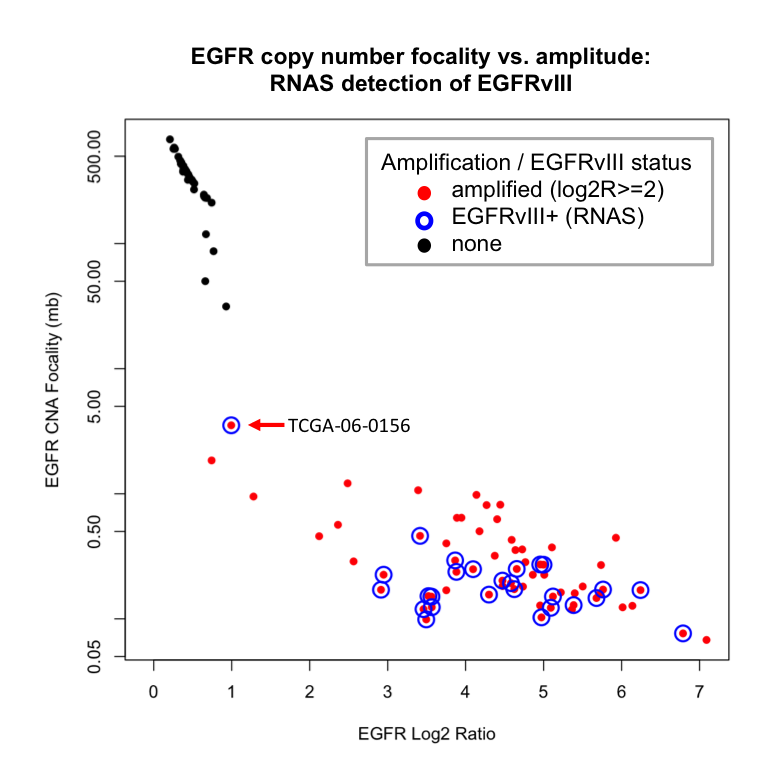

Supplement: Supplementary file 4 — Supplemental Figure S4: EGFRvIII expression, determined from RNA sequencing (RNAS), is restricted to GBMs with focal EGFR amplification. The amplitude and focality of EGFR locus copy number alterations are plotted for cases for which RNA-seq and array-CGH data were available. Focality of CNA is determined based on the extent of Chr7 with log2 ratio at-or-below that of EGFR, using the GTS algorithm previously described [5, 49]. Samples with focal CNA are red (focality < 5mb). Blue denotes cases where EGFRvIII was detected in more than a single read. TCGA-06-0156 was confirmed to harbor high-level EGFR amplification in a subpopulation of tumor cells by FISH [43]. EGFRvIII expression was not found among 52 samples without focal CNA (black dots), while the wild-type junction was read a total of 1789 times. (TIFF 1783 kb) [file 401_2013_1217_MOESM4_ESM.tif]

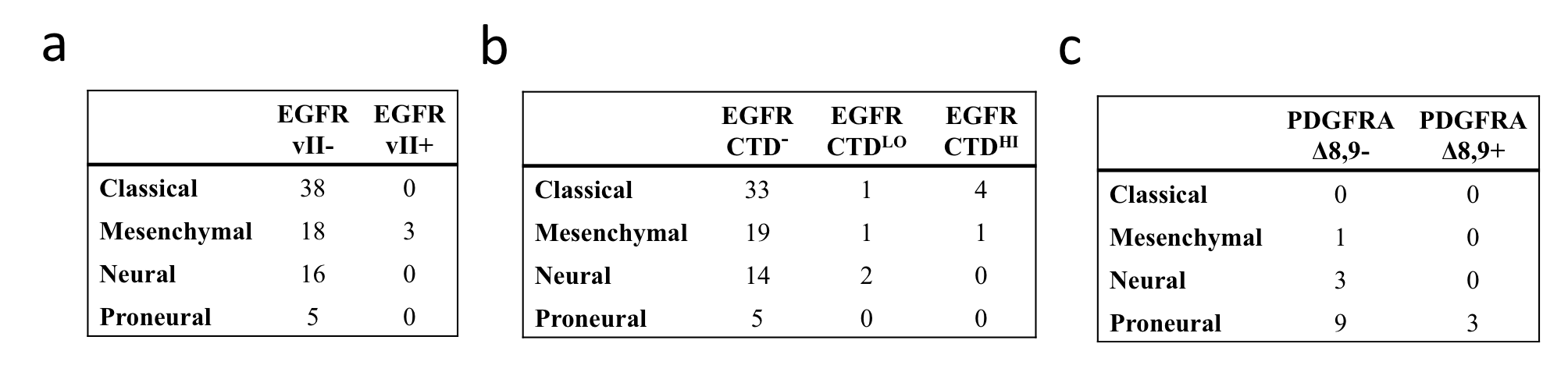

Supplement: Supplementary file 5 — Supplemental Figure S5: Association of transcriptional subclass and (a) EGFRvII, (b) EGFRvV, and (c) PDGFRAD8,9. (TIFF 2774 kb) [file 401_2013_1217_MOESM5_ESM.tif]

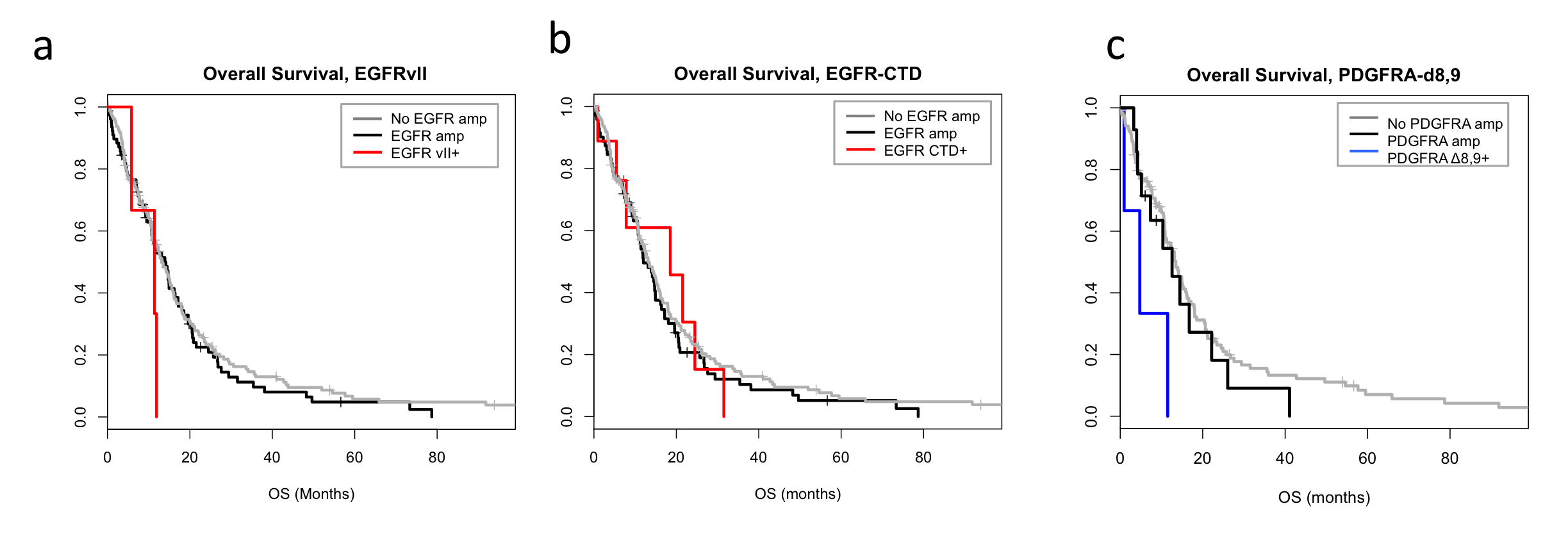

Supplement: Supplementary file 6 — Supplemental Figure S6: Association of overall survival and (a) EGFRvII, (b) EGFRvV, and (c) PDGFRAD8,9. (TIFF 6064 kb) [file 401_2013_1217_MOESM6_ESM.tif]
